# Decline in reported AIDS cases in Brazil after implementation of the test and treat initiative

**DOI:** 10.1186/s12879-019-4018-z

**Published:** 2019-07-04

**Authors:** Gerson Fernando Mendes Pereira, Meritxell Sabidó, Alessandro Caruso, Adele Schwartz Benzaken

**Affiliations:** 1 Department of STI, AIDS and Viral Hepatitis, Secretary for Health Surveillance, Ministry of Health Brazil, Brasília, DF Brazil; 2 TransLab, Department of Medical Sciences, Universitat de Girona, Catalonia, Spain; 3 CIBER Epidemiology and Public Health (CIBERESP), Madrid, Spain

**Keywords:** AIDS, Mortality, Epidemiology, Trends, Brazil

## Abstract

**Background:**

To evaluate the trends in AIDS detection and the AIDS-attributed death rate in Brazil between 2007 and 2015 and to determine the population characteristics associated with AIDS detection.

**Methods:**

Surveillance data including demographics, mode of HIV transmission, AIDS cases, and AIDS-attributed deaths were analysed. A logistic regression model was fitted to assess the trends in AIDS cases by mode of HIV transmission throughout the study period. A segmented Poisson regression model was used to assess changes in the trends of AIDS detection and mortality rates before (2007–2014) and after (2015) the introduction of the Test and Treat Initiative.

**Results:**

In the segmented Poisson regression, the decline in the AIDS detection rate from 2007 to 2014 was 2.0% annually (i.e., the odds ratio (OR) for annual decline was 0.98 (95% [CI: 0.98–1.00, *p*-value < 0.001). The observed AIDS detection rate in 2015 was 7.7 cases per 100,000, which was 60% lower than the regression estimate of 19.8 for the hypothetical absence of the Test and Treat Initiative. The reduction in AIDS-attributed deaths from 2007 to 2014 was 8.0% annually (i.e., the OR for annual decline was 0.92 (95% [CI: 0.91–0.95, *p*-value < 0.001). The observed AIDS mortality rate in 2015 was 0.49 cases per 100,000, which was 73% lower than the regression estimate of 2.1 for the hypothetical absence of the Test and Treat Initiative in 2015.

**Conclusions:**

Our results reveal a fairly stable AIDS detection rate from 2007 to 2014, with a sudden significant drop in 2015. This drop may be related to the increasing trends in rapid testing, the number of new HIV diagnoses, the number of patients on antiretroviral therapy (ART), and a median CD4 count at ART enrolment. Further evaluation of the effects of the Test and Treat Initiative on AIDS diagnosis and mortality is needed and must be strengthened with additional data from subsequent years.

## Background

Approximately 42% of the people living with HIV/AIDS (PLWHA) in Latin America can be found in Brazil [1], with 842,710 AIDS cases registered as of June 2016 [2]. An estimated 32,321 new HIV cases were reported in 2015, which corresponds to a rise of 81% since 2007 (6152) [2].

The reported number of AIDS deaths in Brazil has remained stable from 11,020 cases in 2004 to 12,298 cases in 2015. The AIDS mortality rate has oscillated from 5.9 AIDS-related deaths per 100,000 persons in 2006 to 5.6 per 100,000 persons in 2015, although the trends present regional variations [2].

Brazil HIV/AIDS epidemic scenario concentrates in key groups and is mainly spread by sexual transmission. The country reported an HIV prevalence of 0.4% among adults in 2010. Nevertheless, the prevalence was higher in other groups such as men who have sex with men (MSM) (18.4%) [3], sex workers (5.3%), and drug/alcohol users (5.9%) [4, 5]. The majority of AIDS cases are men, with a sex ratio of 1.7 to one in 2012. Amongst men, heterosexual transmission increased in the 1990s, but MSM still account for the majority of AIDS cases (36.5%) occurring amongst males [2].

By age, most are AIDS cases are in those 25 to 49-years old. The age group 13–19 years is the only one with more women than men affected by AIDS [2]. One-third of AIDS cases hit those with incomplete primary schooling. The risk of HIV/AIDS presents a geographical gradation, with southern states having a larger proportion of PLWHA [2].

Brazil implemented the Test and Treat Initiative following the release of the Clinical Protocol and Therapeutic Guidelines for HIV Infection Management in Adults (CPTG) in 2013 [6]. After adopting the protocol, the percentage of PLWHA who were treated with antiretroviral therapy (ART) increased from 44% in 2012 to 55% in 2015, resulting in 455,000 PLWHA undergoing ART in 2015 [7]. It should be noted that the ART eligibility criteria have evolved over time. In 2001, ART was initiated in patients with CD4 counts < 350 cells/mm^3^, regardless of symptoms or viral load [8]. ART initiation criteria for adult and adolescent patients consisted of a CD4 count ≤500 cells/mm^3^ until 2013 and initiation regardless of CD4 count thereafter [9]. The expansion of ART eligibility will help meet the target of 90% of patients receiving ART by 2020. Although access to ART has expanded in recent years, only 80% of HIV-infected people who knew their HIV status were receiving treatment at the end of 2015 [10].

To accelerate efforts towards ending the AIDS epidemic, Brazil adopted the 90–90-90 and 95–95-95 targets in 2014. The 90–90-90 target calls for 90% of the people living with HIV to be diagnosed, 90% of the diagnosed people to be receiving sustained ART, and viral suppression in 90% of patients on ART by 2020 [7]. The 90–90-90 and the subsequent 95–95-95 by 2030 targets translate into 84 and 54% of patients being diagnosed and virally supressed amongst PLWHA, respectively [10]. Critical efforts to achieve these targets include scaling up access to prevention, diagnosis, and treatment; combating stigma and discrimination; and promoting human rights, particularly to the key populations affected by the epidemic and people living with HIV. These efforts have been mobilized through the Unified Health System (Sistema Único de Saúde - SUS), although civil society organizations have also played a prominent role [7].

Understanding the evolution of AIDS incidence and mortality is relevant to the response to HIV/AIDS. We used data generated from the surveillance system to reconstruct the pattern of AIDS detection and the AIDS-attributed mortality rate in Brazil. In addition, we examined linkages between AIDS detection and sociodemographic factors.

## Methods

### Setting

Brazil has a publicly funded healthcare system based on universal access to health services that fully subsidizes access to medical services, centralized laboratory monitoring, and access to ART. ART is distributed centrally, and patients primarily attend tertiary care hospitals in capital cities.

### Data sources

We analysed AIDS cases and related variables reported to the Brazilian surveillance system from 2007 to 2015. Data was obtained from several electronic surveillance subsystems: 1) the Notifiable Disorders Information System [*Sistema de Informação de Agravos de Notificação* (SINAN)]: AIDS cases, epidemiological characteristics (eg, sex, age); 2) the Mortality Information System [*Sistema de Informaçoes sobre Mortalidade (SIM)*]: death information such as cause of death, the date, and the municipality of death were recorded from the declaration of death document; 3) the Logistic Treatment Control System [*Sistema de Controle Logistico de Medicamentos (Siclom)*]; and 4) the Laboratory Testing Information System [*Sistema de Informa*çã*o de Exames Laboratoriais (Siscel)*].

### Study population

The national AIDS definition was used to report AIDS cases to the national surveillance system [11]. This definition was revised in 2003 to include a CD4 cell count less than 350 cells/mm^3^. Until 2003, HIV testing was performed by enzyme-linked immunosorbent assay (ELISA). All positive results were confirmed with a second ELISA and Western blot. After 2003, two rapid tests were performed sequentially on different finger prick specimens. If the results between the two tests were discordant, the testing was repeated. We included cases that were at least 15 years of age.

### Data collection

Age, sex, mode of HIV exposure, education level, vital status, race/colour, and date of AIDS diagnosis were collected. The potential mode of HIV transmission was determined based on several variables: 1) sexual activity of the following categories: sex with men, sex with women, sex with men and women, no sexual transmission; 2) intravenous drug users (IDUs) (Yes/No/Unknown); 3) haemophilia treatment or transfusion (Yes/No/Unknown); 4) vertical transmission (Yes/No/Unknown); and 5) biological material accident with seroconversion within 6 months (Yes/No/Unknown). Death was determined based on the recorded date of death. Key indicators related to the cascade of HIV care, such as the number of rapid tests, the number of new HIV diagnoses, the number of PLWHA on ART, and the median CD4 count at ART enrolment, were obtained from national programme data [10].

### Statistical analysis

Stata V11.0 (StataCorp LP, College Station, TX, USA) was used for the analysis. Data was described using frequencies or percentages or means with standard deviations (SDs).

Population estimates were obtained for the years 2007–2015 to calculate mortality and AIDS rates (per 100,000). These estimates were obtained from the Institute of Statistics in Brazil (IBGE – Instituto Brasileiro de Geografia e Estatística). Trends in the AIDS detection rate were evaluated by sex and age group, and trends in the AIDS detection rate and AIDS-attributed death rates were evaluated by sex. Trends in sociodemographic characteristics, mode of HIV exposure, and death were evaluated according to the proportion of AIDS diagnoses over time. The analysis of the mode of HIV exposure was conducted separately for heterosexuals, homosexual/bisexual men, IDUs, vertical transmission, and others. Homosexual/bisexual men were defined as men who reported having sex with men or with men and women as the potential exposure route. IDUs were participants who reported this as the potential transmission route. Participants who reported sexual intercourse with the other sex as the potential exposure route were classified as heterosexuals; homosexual/bisexual women were included in this group. Homosexual/bisexual men and heterosexuals with a history of being IDUs were part of the homosexual/bisexual men, heterosexual, and IDU analyses. The “others” mode of transmission included transfusion, biological accidents, and haemophilia.

The Test and Treat Initiative was adopted in December 2013 and implemented during 2014. Changes in the trends of the AIDS detection rate and the AIDS mortality rate after the introduction of the Test and Treat Initiative in relation to the period before the intervention (2007–2014) were assessed through a segmented regression Poisson model of an interrupted time series. The year 2015 was used as the interruption year for the interrupted trend regression analysis [12]. A continuation of the pre-intervention trend over time (2007–2014) was estimated throughout 2015 as if Test and Treat had not been implemented. A *p*-value < 0.05 was considered significant.

### Ethics

This analysis is part of the Ministry of Health’s strategic plan to enhance HIV testing and was not assessed by an ethical committee board. In Brazil, according to law CFM n° 1.865/96, authorization or consent from parents or a responsible person is not required to perform an HIV test on individuals over 12 years of age if the test is voluntary and consent is provided by the adolescent, provided that the adolescent has the capacity to evaluate their problem and take actions accordingly. Administrative permission to access the raw data of the datasources used were obtained from the Department of STI, AIDS and Viral Hepatitis of the Brazilian Ministry of Health.

## Results

### Sample characteristics

From January 2007 to December 2015, a total of 329,154 AIDS cases were registered in the national surveillance system. The mean patient age was 38.0 years (SD: 11.5), and 63.2% were men. Table 1 describes the study population in terms of demographic characteristics, mode of HIV transmission, and death, stratified by sex.

**Table 1:** Characteristics of cumulative known HIV cases (alive and death) by sex, Brazil, 2007–2015

	Female (*n* = 120,974)		Male (*n* = 208,002)	
	n	%	n	%
Age, years (*n* = 328,372)				
15–24	12,944	10.7	21,184	10.2
25–34	37,592	31.2	68,413	32.9
35–44	35,954	29.8	62,956	30.3
45–54	22,482	18.6	37,572	18.1
≥ 55	11,671	9.7	17,604	8.5
Mode of HIV exposure				
Heterosexual (*n* = 328,976)	73,417	60.7	70,291	33.8
Homosexual/bisexual men (*n* = 208,002)	NA	NA	52,605	25.3
IDU (*n* = 198,452)	1801	2.5	7484	6.0
Vertical transmission (*n* = 225,593)	616	0.8	748	0.5
Others (*n* = 328,976)	678	0.6	863	0.4
Level of education (*n* = 149,572)				
Incomplete primary school	2120	3.8	2730	2.9
Incomplete secondary school	13,011	23.6	18,143	19.2
Incomplete post-secondary school	23,937	43.4	35,731	37.9
≥ Complete post-secondary school	16,115	29.2	37,785	40.0
Race/colour (*n* = 199,044)				
White	31,983	45.9	63,455	49.1
Black	8867	12.7	13,037	10.1
Asian	335	0.5	678	0.5
Brown (Pardo)	28,294	40.6	51,759	40.0
Indigenous	253	0.4	383	0.3
Death, any cause (*n* = 234,809)	12,973	15.7	28,208	18.5

Heterosexual intercourse was reported as the mode of transmission in 60.7 and 33.8% of AIDS cases in women and men, respectively. Overall, 25.3% of AIDS cases were reported in homosexual/bisexual men. AIDS cases due to IDU represented 2.5% of cases in women and 6.0% of cases in men. In both sexes, a small proportion of cases (< 1%) occurred via vertical transmission and other modes of HIV exposure. Regarding the level of education, the highest proportions of cases were found in women with incomplete post-secondary education (43.4%) and in patients with at least a post-secondary education (40.0%). The highest proportion of AIDS cases was found in white people (45.9% in women; 49.1% in men), followed by brown people (40.6% in women; 40.0% in men). Death occurred in 15.7% of female cases and 18.5% of male cases.

### Changes in the AIDS detection and AIDS-attributed death rates over time

**Fig. 1:**
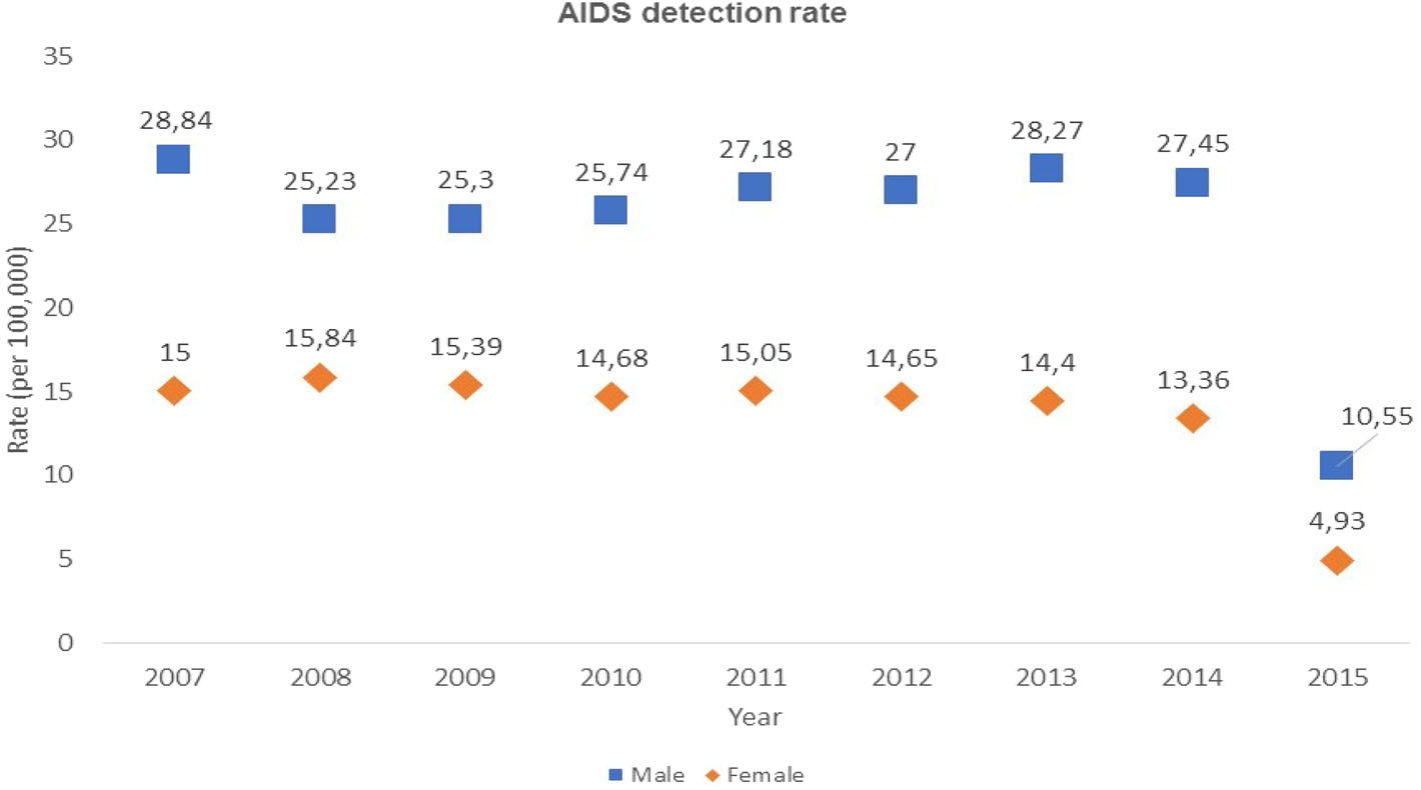
Trends in AIDS detection rates by sex and by treatment status, Brazil 2007–2015

The AIDS detection rate (per 100,000) before the Test and Treat Initiative (2007–2014) was 20.45; right after its implementation (2015), this rate was 7.70 (Fig. 1), which represents an approximately 62% crude decrease. In the segmented Poisson regression, the decline over 2007–2014 was 2.0% annually (i.e., the odds ratio (OR) for annual decline was 0.98 (95% [CI: 0.98–1.00, *p*-value < 0.001), and the predicted AIDS detection rate in 2015 without implementation of the Test and Treat Initiative in 2015 would have been 19.8. Thus, the estimated drop in 2015 (adjusted for the pre-2015 trend) was (1–7.7/19.8) = 60%.

**Fig. 2:**
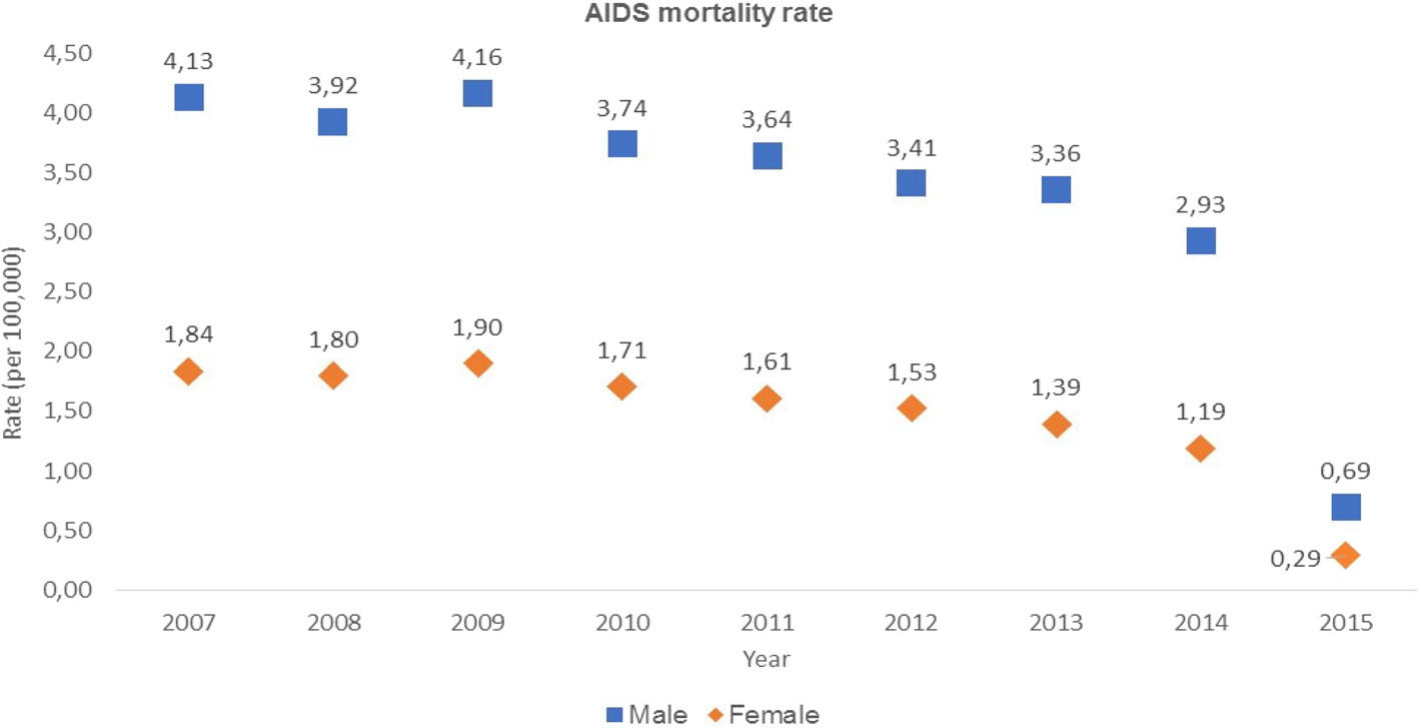
Trends in AIDS-attributed deaths rates by sex and by treatment status, Brazil, 2007–2015

The mortality rate was 2.32 (per 100,000) before the Test and Treat Initiative (2007–2014); right after its implementation (2015) (Fig. 2), the rate was 0.49, which represents a crude decline of 79%. In the segmented Poisson regression, the decline over 2007–14 was 8.0% annually (i.e., the OR for annual decline was 0.92 (95% [CI: 0.91–0.95, *p*-value < 0.001), and the predicted AIDS mortality rate in 2015 without implementation of the Test and Treat Initiative in 2015 would have been 2.1. Thus, the estimated drop in 2015 (adjusted for pre-2015 trend) was (1–0.49/2.2) = 73%.

### Modes of HIV exposure amongst reported AIDS cases

The proportions of reported AIDS cases amongst heterosexuals were stable in the first few years and then declined in men after 2010 and in women only after 2013 (Table 2). The proportion of cases reported in homosexual/bisexual men increased over time but declined in the final 2 years. The proportion of AIDS cases caused by IDU decreased in both sexes, although the decline was more pronounced in men (from 9.0% in 2007 to 3.5% in 2015) than in women (from 3.1% in 2007 to 2.2% in 2015). An increasing trend in vertical transmission was observed for the proportion of AIDS cases in both sexes. A decrease was observed in the proportion of reported AIDS cases caused by other modes of transmission (less than 1% in both sexes).

**Table 2:** Mode of HIV exposure amongst reported AIDS cases by sex, Brazil, 2007–2015 (%)

	2007	2008	2009	2010	2011	2012	2013	2014	2015
Female									
n	14,474	15,350	15,023	14,326	14,826	14,526	14,298	13,259	4892
Heterosexual	59.0	59.7	61.3	63.6	62.8	63.2	62.4	57.9	47.1
IDU	3.1	2.8	2.5	2.6	2.4	2.6	1.9	2.3	2.2
Vertical transmission	0.5	0.7	0.7	0.9	0.50	0.7	0.8	0.9	1.2
Others	0.9	0.7	0.7	0.50	0.5	0.4	0.5	0.4	0.4
Male									
n	22,222	23,534	23,835	24,087	25,633	25,685	26,884	26,094	10,028
Heterosexual	35.0	34.5	35.0	35.9	35.0	34.7	33.3	30.9	25.3
Homosexual/bisexual men	21.4	21.8	23.5	25.3	26.5	28.1	27.8	27.6	23.1
IDU	9.0	7.9	7.3	6.0	5.7	4.7	4.5	4.0	3.5
Vertical transmission	0.3	0.4	0.4	0.5	0.5	0.5	0.6	0.7	0.8
Others	0.6	0.6	0.5	0.4	0.3	0.4	0.33	0.3	0.3

### Key indicators of the cascade of HIV care

Table 3 displays key indicators related to the cascade of HIV care over time. The number of PLWHA on ART increased from 181,000 people in 2007 to more than 455,000 people in 2015. The median CD4 of PLWHA enrolling in ART also increased from 390 cells/mm^3^ in 2013 to 416 cells/mm^3^ in 2015. The number of rapid tests and new HIV diagnoses also increased over time, with an acceleration beginning in 2013.

**Table 3:** Key indicators related to the cascade of HIV care over time, Brazil, 2007–2015 (%)

	2007	2008	2009	2010	2011	2012	2013	2014	2015
HIV rapid tests distributed	1,225,175	1,754,335	2,446,380	1,680,940	2,307,615	3,750,400	4,729,485	6,492,105	8,533,270
New HIV diagnoses	6152	6654	7126	8316	9863	11,223	16,331	26,277	32,321
PLWHA alive on ART^a^	181,000	193,000	231,000	257,000	284,000	313,000	355,000	410,000	455,000
Median CD4 count^b^	NA^c^	NA^c^	361	364	374	379	390	404	416
New AIDS cases	36,632	38,813	38,812	38,340	40,395	40,160	41,125	39,330	14,941

^a^ Between 1999 and 2008, the number of people living with HIV (PLWHA) in ART was estimated considering the number of dispensations performed in December of each year. As of 2009, PLWHA in ART are those that have had at least one ARV dispensation in the last 100 days of the year. ^b^ Median CD4 count (cell/mm^3^) of PLWHA enrolling in ART. ^c^ NA: Data not available because the Logistic Treatment Control System [Sistema de Controle Logistico de Medicamentos (Siclom)] was in an implementation stage, which influenced the data quality

## Discussion

This study provides an overview of 9-year trends in the detection of AIDS cases in Brazil. Our findings show that the AIDS detection rate remained stable from 2007 to 2014 but displayed a significant decline the following year (2015). This decline was observed in both sexes. This change might be explained by the adoption of the Test and Treat Initiative in December 2013 and its subsequent implementation in 2014, offering early treatment to all HIV-positive adults, regardless of their CD4 cell counts or viral load; in this context, treatment is also used as a form of prevention (TasP).

TasP is likely the major driver of the reductions in AIDS morbidity and mortality. Amongst treated HIV-infected patients, ART leads to immune reconstitution, preventing the emergence of AIDS events and death [13–15].

Brazil adopted the Test and Treat Initiative two years ahead of the standard WHO “test and wait”, which was modified in 2015. The early adaption in Brazil correlated with increases in HIV diagnoses and patients receiving treatment; therefore, the initiative might be a major driver behind the observed reduction in AIDS cases. The number of HIV rapid tests increased over time, with acceleration from 2012. Likewise, the number of new HIV diagnoses and the number of PLWHA on ART (reflecting improved survival) showed increasing trends with acceleration from 2013 [10]. As emphasized by the WHO’s 2013 consolidated guidelines, early ART initiation is essential to leverage the full range of benefits that ART offers [16]. Brazil has displayed a shift towards earlier initiation of ART, as demonstrated by the increase in the proportion of patients with pre-ART CD4 cell counts above 500 cells/mm^3^ (from 17% in 2013 to 37% in 2016) [10]. The median CD4 of PLWHA enrolling in ART has also increased over time with acceleration after 2013 [10]. This increase suggests that the Test and Treat Initiative was effective.

The significant decrease in AIDS-attributed deaths in the final year might also be attributed to the TasP strategy [13–15]. Scaling up ART is strongly and significantly associated with population-level decreases in all-cause mortality amongst HIV-infected individuals [17]. It is unlikely that pre-exposure prophylaxis (PrEP) plays a role in the results of this study. In Brazil, the first steps for offering PrEP on a large scale started in 2013, when the Ministry of Health funded 5 separate small-scale PrEP demonstration projects [18]. PrEP distribution for HIV prevention in key higher-risk population groups throughout 35 sites across the country only started in January 2018. As of April 2018, the estimated number of current PrEP users was 1000–1500, and it is estimated that in this first year, 9000 patients will benefit from PrEP.

Brazil has adopted combination prevention, which is a strategy that involves post-exposure prophylaxis (PEP); PrEP; mass campaigns to encourage condom use and testing; distribution of condoms; and specific HIV prevention actions with key populations [7]. Indeed, increasing access to prevention and diagnosis, particularly for the key populations affected by the epidemic, was key to the success of the Test and Treat strategy. Standing out amongst these efforts is the programme Viva Melhor Sabendo (*Live Better Knowing*), which was implemented in 2013 [7]. The programme consists of outreach peer prevention intervention that offers rapid oral fluid HIV testing in the community [19]. The Department of Sexually Transmitted Infections, AIDS, and Viral Hepatitis has worked closely with civil society organizations to implement this programme. This close collaboration has been also essential to link HIV cases detected with the health system [19]. Between January 2013 and April 2016, 43,358 participants were tested, and roughly half (52.1%) were tested for HIV first time ever [20]. These results suggest that the programme is reaching people with low access to testing and who might belong to high-risk groups [20]. However, efforts are still needed to promote testing, particularly amongst the most vulnerable groups.

A major challenge for the country will be expanding periodic HIV testing, specifically by targeting the most at-risk populations. To expand ART for HIV-infected individuals and increase survival, estimates of HIV incidence must be incorporated into HIV/AIDS surveillance activities [21].

The period of 2007 to 2013 displayed an increasing trend in the AIDS detection rate in men. When assessed by education, there was found to be an increase only in well-educated people. Changing patterns of risk behaviours could have influenced our results. Notably, there was a significant increase in diagnosed AIDS cases amongst homosexual/bisexual men in Brazil; this population is driving the HIV epidemic in the south of the country. It is likely that the intense macho culture that prevails discourages homosexual/bisexual men from identifying as such and therefore from adopting preventive measures. Brazil began distributing PrEP in January 2018 as part of their combined prevention initiatives [7], and homosexual/bisexual men are a key population that may benefit.

By contrast, AIDS cases in IDUs have significantly decreased over time. In Brazil, IDU has been progressively supplanted by crack cocaine. However, 5% of crack users in Brazil have HIV-AIDS, a rate 12 times higher than that of the general population [7].

AIDS cases amongst indigenous people have remained low and stable. In all age groups, AIDS was more prevalent amongst men than women. The age group most affected was 25- to 44-year-olds.

We have analysed data on AIDS cases reported to the national surveillance system, which is subject to some limitations. We cannot rule out the underreporting of AIDS cases and AIDS-attributed deaths rates, particularly in a context where a substantial number of infected subjects are unaware of their HIV status. Mortality may have multiple causes, and determining the attribution of HIV or other aetiologies is difficult.

## Conclusions

In conclusion, our results show fairly stable levels of AIDS detection rate and of AIDS-attributed deaths over 2007–2014, with sudden significant drops in 2015. The drop in the AIDS detection rate might be related to the scale-up of HIV rapid testing (increasing number, with a more rapid increase beginning in 2012), the increasing trend in the number of PLWA on ART, which reflects improved survival (with a more rapid increase beginning in 2013) and the median CD4 count at ART enrolment (increasing, with a more rapid increase beginning in 2013). Given that the reduction in the number of AIDS diagnoses is apparent for only one year thus far, this observation and its interpretation require further confirmation and must be strengthened with similar analyses using additional years of data (2016 and 2017). Future studies could explore whether this decrease leads to a reduction in HIV transmission at the population level [17].

Brazil must continue expanding ART and achieving universal access to therapy. Although gains in ART coverage have been reported, ART-eligible individuals in northern Brazil are notably less likely to obtain ART than those in southern Brazil. Other improvements, such as improved availability of the combined triple-dose, 3-in-1 medication, and use of the preferred first-line regimen in 2015, might enhance the decreasing trends in the AIDS detection and AIDS-attributed death rates.

## Abbreviations

AIDS: Acquired immune deficiency syndrome
ART: Antiretroviral therapy
CI: Confidence interval
ELISA: Enzyme-linked immunosorbent assay
HIV: Human immunodeficiency virus
HIV/AIDS ᅟ: Human immunodeficiency virus/Acquired immune deficiency syndrome
IBGE: Instituto Brasileiro de Geografia e Estatística (Institute of Statistics in Brazil
IDU: Intravenous drug users
MSM: Men who have sex with men
OR: Odds ratio
PCAP: Knowledge, attitudes, and practices
PEP: Post-exposure prophylaxis
PLWHA: People living with HIV/AIDS
PrEP: Pre-exposure prophylaxis
SICLOM: Logistic Treatment Control System
SIM: Mortality Information System
SINAN: Notifiable Disorders Information System
SISCEL: Laboratory Testing Information System
SUS: Unified Health System (Sistema Único de Saúde)
TasP: Treatment as prevention
